# Modeling Habitat Suitability of Snow Leopards in Yanchiwan National Reserve, China

**DOI:** 10.3390/ani14131938

**Published:** 2024-06-30

**Authors:** Rashid Rasool Rabbani Ismaili, Xiaoxu Peng, Yibin Li, Arshad Ali, Tariq Ahmad, Anees Ur Rahman, Shahid Ahmad, Kun Shi

**Affiliations:** 1 The Wildlife Institute, School of Ecology & Nature Conservation, Beijing Forestry University, Beijing 100083, China; rashid.rri1998@gmail.com (R.R.R.I.); anis9003617@bjfu.edu.cn (A.U.R.); 2 Zhejiang Huadong Forestry Engineering Consulting and Design Corporation, Hangzhou 310019, China; pxx424@foxmail.com; 3 Eco-Bridge Continental, Beijing 100085, China; li.yibin@foxmail.com; 4 Department of Zoology, Malakand University, Chakdara 18800, Lower Dir, Khyber Pakhtunkhwa, Pakistan; arshadkotlai1985@gmail.com; 5 College of Wildlife and Protected Area, Northeast Forestry University, Harbin 150040, China; tariq.zoologist@yahoo.com; 6 School of Ecology and Environment, Hainan University, Haikou 570228, China; 184126@hainanu.edu.cn

**Keywords:** snow leopard, maximum entropy modeling, Yanchiwan National Nature Reserve, species conservation, environmental impact

## Abstract

**Simple Summary:**

This study was conducted in the Yanchiwan National Nature Reserve in Gansu, China, using data collected between 2019 and 2022 to determine habitat suitability for snow leopards. Employing the MaxEnt model, this research examined key environmental parameters like altitude, soil type, human footprint, forest cover, prey availability, and human disturbances. The findings show the importance of these factors in determining snow leopard distribution, with particular emphasis on how human activities influence habitat suitability. This study stresses the importance of conservation methods that focus on these components to sustain the survival of snow leopards in the region and protect this endangered species. Furthermore, the use of advanced modeling approaches like MaxEnt improves our understanding of habitat dynamics and serves as the foundation for focused conservation policies that can be applied to similar wildlife contexts around the world. This method guarantees that conservation efforts are guided by precise, data-driven insights, allowing for more effective management and protection of snow leopard habitats in the face of growing environmental and anthropogenic challenges.

**Abstract:**

Snow leopards (*Panthera uncia*) are elusive predators inhabiting high-altitude and mountainous rugged habitats. The current study was conducted in the Yanchiwan National Nature Reserve, Gansu Province, China, to assess the habitat suitability of snow leopards and identify key environmental factors inducing their distribution. Field data collected between 2019 and 2022 through scat sampling and camera trapping techniques provided insights into snow leopard habitat preferences. Spatial distribution and cluster analyses show distinct hotspots of high habitat suitability, mostly concentrated near mountainous landscapes. While altitude remains a critical determinant, with places above 3300 m showing increased habitat suitability, other factors such as soil type, human footprint, forest cover, prey availability, and human disturbance also play important roles. These variables influence ecological dynamics and are required to assess and manage snow leopard habitats. The MaxEnt model has helped us to better grasp these issues, particularly the enormous impact of human activities on habitat suitability. The current study highlights the importance of altitude in determining snow leopard habitat preferences and distribution patterns in the reserve. Furthermore, the study underscores the significance of considering elevation in conservation planning and management strategies for snow leopards, particularly in mountainous regions. By combining complete environmental data with innovative modeling tools, this study not only improves local conservation efforts but also serves as a model for similar wildlife conservation initiatives around the world. By understanding the environmental factors driving snow leopard distribution, conservation efforts can be more efficiently directed to ensure the long-term survival of this endangered species. This study provides valuable insights for evidence-based conservation efforts to safeguard the habitats of snow leopards amidst emerging anthropogenic pressure and environmental fluctuations.

## 1. Introduction

The snow leopard (*Panthera uncia*) is an endangered big cat of the alpine region. It is distributed in 12 countries, including China, Russia, Nepal, Mongolia, and other alpine areas in Central Asia [1]. Snow leopards occupy the top of the food chain and their survival is a good reflection of the health of the entire mountain ecosystem [2]. Habitat, defined as the natural environment where species reside and flourish, encompasses areas that offer vital resources such as food, shelter, and breeding grounds. One of the best ways to protect species is to protect their habitats, especially with reasonable planning of protected areas and in situ conservation policies [2]. China is one of the countries with the largest distribution of snow leopards in the world. Early research shows that the suitable habitat areas for snow leopards in the world is about 3.02 million km^2^, of which 60% are in China, about 1.82 million km^2^, accounting for 18.7% of China’s land area, in Qinghai, Tibet, Xinjiang, Gansu, Sichuan [3]. Nearly a quarter of all mammal species worldwide are threatened with extinction due to habitat fragmentation [4]. Conducting habitat suitability and predicting species habitat distribution are significant for evaluating the current status of such habitats. This understanding offers important theoretical support for conservation and management initiatives. Most studies of snow leopards focus on structure, genetics, and taxonomy; however, there is an evident lack of ecological studies. The research areas on the habitat suitability of snow leopards are also concentrated in Qinghai, Xinjiang, Sichuan, and other places [5,6]. The research on snow leopards in Gansu province still needs further in-depth research. Schaller [7] recorded as many as 650 snow leopards in the fringes of Qinghai and Gansu provinces. During the 2018 monitoring campaign, researchers also discovered the distribution of snow leopards in the Yanchiwan National and Nature Reserve in Gansu, China [8]. However, there is no research on the habitat suitability of snow leopards in Gansu Province and the impact of their living environment on the distribution of snow leopards.

Despite snow leopards’ widespread distribution over high-altitude landscapes, research focused on the Yanchiwan National Nature Reserve provides a unique chance to enhance our understanding of their habitat preferences in a less-studied but ecologically significant area. This study attempts to address this critical knowledge gap by focusing on a comprehensive habitat evaluation in the Yanchiwan reserve, resulting in a more nuanced understanding of snow leopard habitats. Such discoveries are critical for shaping local conservation policies and can serve as a model for similar studies in other understudied places throughout the world.

The MaxEnt model has gained popularity in recent years for species habitat evaluation and geographic distribution prediction because of its capacity to manage data with fewer inputs while maintaining good prediction accuracy [9,10]. MaxEnt, or maximum entropy modeling, combines maximum entropy principles with ecological niches to estimate species distribution based on known occurrences and environmental variables [11,12]. This model is very useful for conserving endangered species, such as the Amur tiger (*Panthera tigris altaica*) in Russia’s Far East, where it has been used to anticipate probable distribution ranges and inform conservation measures [13]. Similarly, in China, which accounts for roughly 60% of worldwide snow leopard habitat, the MaxEnt model is critical for addressing research gaps in areas such as Gansu Province, where data on habitat suitability are lacking [14,15]. This work uses the MaxEnt model to assess the habitat suitability of snow leopards in Yanchiwan National Nature Reserve, with the goal of improving conservation planning and addressing habitat fragmentation [16,17]. By projecting accurate species distributions and identifying crucial habitats, the model aids in the creation of protected areas and contributes to overall ecological balance and conservation efforts.

## 2. Materials and Methods

### 2.1. Study Area

**Figure 1:**
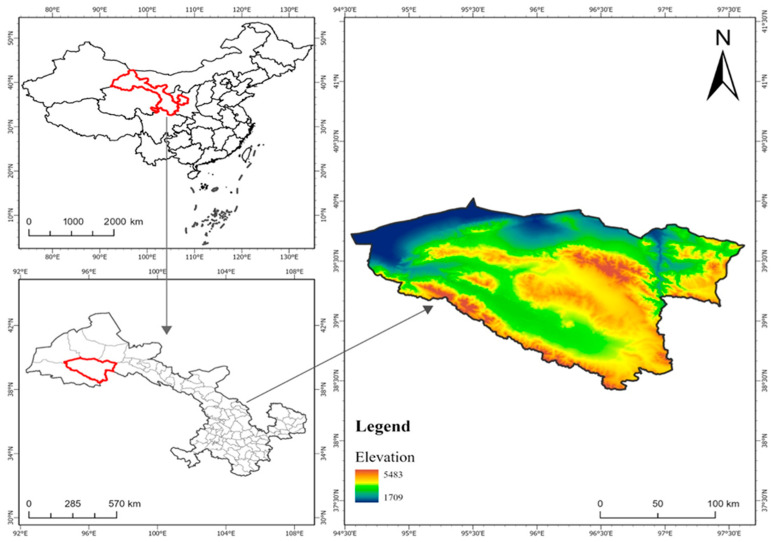
Map of the study area, representing Yanchiwan National Nature Reserve.

The Yanchiwan National Nature Reserve is located at the western end of Qilian Mountains, in the southeast of Subei Mongolian Autonomous County, with an average altitude of more than 3000 m (Figure 1). It is a Wildlife-type nature reserve with a complex terrain of overlapping mountains and rivers, juxtaposition of canyons, and alternating basins. The terrain slopes from east to west, and from the northwest to the southeast, it encompasses four parallel mountain ranges: Daxue Mountain, Yema Nanshan, Tuolai Nanshan, and Danghenan Mountain. These ranges are the sources of the Shule River, Dang River, and Yulin River [18,19]. The protected area is divided into three functional areas: the core area, the buffer area, and the experimental area. The core area is 3508.87 km^2^, accounting for 25.73% of the total area of the protected area; the buffer area is 2821.03 km^2^, accounting for 20.68% of the total area of the protected area; and the area of the experimental area is 7309.69 km^2^, accounting for 53.59% of the total protected area [20]. The topographic features of the reserve are very rich, including basins, valleys, canyons, wetlands, etc. Yanchiwan National Nature Reserve, as a gathering place for fragile ecosystems and rare wild animals and plants, should receive more attention and protection. In recent years, the continuous changes in climate and human activities have directly or indirectly changed the energy flow and material circulation between different landscape mosaics in the Yanchiwan National Nature Reserve, resulting in changes in the ecological processes between landscape elements, and ultimately affecting the ecosystem function of protected areas [20].

### 2.2. Snow Leopard Species Distribution Data Collection

Data on snow leopard distribution were collected from 2019 to 2022 in the Yanchiwan National Reserve. The data were gathered following by the two methods, i.e., scat sampling and camera trapping. This dual-method approach allows for cross-verification of the presence of snow leopards, enhancing the reliability of the distribution data collected. We collected data in the Yema Nanshan, Shule Nanshan, Yuerhong, and Dangnan Mountains of the Yanchiwan National Nature Reserve, building on and expanding Wang’s [21] findings. While Wang’s study examined snow leopard habitats throughout the larger Qilian Mountain National Park, which includes parts of the Yanchiwan National Nature Reserve, our research focuses on these four mountains and uses a different methodological approach to assess habitat suitability. This allows for a more in-depth understanding of habitat dynamics within the reserve’s geographically diverse biological zones.

This research utilized advanced spatial and temporal analysis tools to evaluate habitat suitability and connectivity, identifying key areas that support viable snow leopard populations. The methodologies and insights gained from Wang’s study guided our selection, ensuring that our chosen mountains have documented significance in terms of ecological value and potential for conservation impact.

### 2.3. Spatial Distribution Analysis for Snow Leopard Habitat Suitability

To supplement MaxEnt’s prediction power, Spatial Distribution and Cluster Analyses were added to provide more detailed, actionable data. These methods give complementary layers of information that, when combined, create a more complete picture of habitat suitability and variability across the reserve, influencing both strategic planning and immediate conservation efforts. The spatial distribution research was primarily focused on identifying microhabitats with a higher frequency of snow leopard activity, using both direct observations and indirect data obtained via camera traps.

### 2.4. Cluster Analysis for Snow Leopard Habitat Suitability

The methodology for this study involved conducting a cluster analysis to identify distinct habitat types within the Yanchiwan National Nature Reserve based on environmental characteristics and habitat suitability for snow leopards. The analysis was performed using the R Program version (4.3.2). The data included geographic coordinates, altitude, temperature, vegetation type, and human proximity, which were selected as key indicators of habitat suitability. This analytical technique assisted in identifying parts of the reserve that are crucial to snow leopard conservation and require specific management actions. We used a multimodal strategy to select the most significant environmental variables for snow leopards in the Yanchiwan National Nature Reserve. The rationale for selecting these indicators was as follows:

Ecological Relevance: Each indicator was assessed for its direct or indirect impact on the life cycles and behavior of snow leopards. Altitude, vegetation type, and prey availability were included as they have been shown to influence snow leopard distribution and survival tactics.

Data Availability: We chose indicators for which accurate, high-resolution spatial data were available. This ensured that our habitat suitability models were based on reliable and comprehensive environmental data.

Proven Impact in Similar Studies: After reviewing significant literature on habitat modeling for snow leopards and other large predators, we identified variables that were consistently associated with habitat preferences and species distribution. Studies by [14,22] provided benchmarks for selecting characteristics such as the human footprint and soil type, both of which have been shown to have a major impact on carnivore habitats.

Statistical Robustness: The selected indicators were examined for statistical significance in preliminary models to ensure that they made substantial contributions to the models’ predictive accuracy.

Management Applicability: Indicators were chosen based on their utility in conservation management, with an emphasis on factors that local wildlife agencies can monitor and manage. By combining these criteria, we created a robust collection of environmental factors that provide a comprehensive view of habitat suitability for snow leopards in the area.

Before the cluster analysis, the data were standardized to ensure comparability across variables with different scales. Hierarchical clustering with Ward’s method was used to group areas within the reserve, using the Euclidean distance metric to measure similarity between data points. The “elbow method” was applied to determine the optimal number of clusters, and the dendrogram was cut at this point to create distinct clusters. To visualize the results, a scatter plot was created using ggplot2, an R package (version 4.3.2) for data visualization, showing the spatial distribution of the clusters across the reserve.

### 2.5. Temporal Trends in Habitat Suitability for Snow Leopards

This section of the study examined changes in habitat characteristics such as vegetation cover and human disturbance over time in order to better understand their long-term effects on snow leopard habitat suitability. By following these characteristics annually, we discovered trends that could anticipate future changes in habitat suitability, which is critical for long-term conservation plans.

### 2.6. Collection of Scat Samples of Snow Leopards

To collect scat samples of the snow leopard, a line transect of one km in length and 200 m wide was laid in four outermost sampling sites, ensuring geographic diversity. A total of 50 line transects were surveyed across the study area. The scat samples were placed in silica gel immediately upon collection in the field; the collected scats were then stored in 50 mL polypropylene centrifuge tubes containing 30 mL silica gel upon arrival at the lab and allowed to remain in the silica gel for 48 h to ensure proper desiccation and preservation before further analysis. They were immediately placed in a −20 °C freezer. After completion of scat samples of snow leopards, DNA was extracted from each of the collected samples, using QiAamp DNA kit followed by species identification using polymerase chain reaction (PCR) with two primers Cytb and ATP6 (Zhang et al., 2019 [15]). This research revealed information about the genetic variety of the snow leopard population in the reserve, which is crucial for understanding breeding patterns and population health.

FARRELL F—TATTCTTTATCTGCCTATACATACACG

FARRELL R—AAACTGCAGCCCCTCAGAATGATATTTGTCCTCA

ATP6 primers

DF3—AACGAAAATCTATTCGCCTCT

DR1—CCAGTATTTGTTTTGATGTTAGTTG

#### Camera Trap Data Collection

A total of 20 motion-triggered camera traps were installed across the study area. These cameras were strategically positioned at key locations known to be used by snow leopards, comprising identified migration routes, potential hunting grounds, and areas with prior evidence of snow leopard presence. These cameras were critical not just for detecting snow leopards, but also for tracking their behavioral patterns and interactions with the ecosystem.

**Figure 2:**
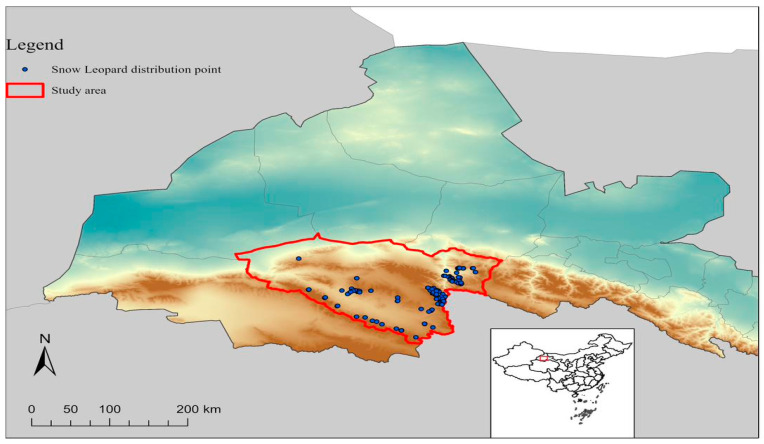
Map of study area showing the occurrence of snow leopards in Yanchiwan National Nature Reserve.

Each camera was set to record for 24 h per day regularly throughout the study period, with batteries and memory cards changed regularly to guarantee uninterrupted data collection. The positioning of the cameras remained consistent throughout the study to maintain reliability and maximize coverage of the target areas. No significant changes in camera placement were made during the study to minimize any bias in data collection. On the other hand, for secondary data related to climatic factors, from various official websites (http://www.forestry.gov.cn/, http://www.mee.gov.cn/, accessed on 20 January 2022) of the forestry and ecology department were reviewed. A diagram of the occurrence of snow leopards is shown in (Figure 2).

### 2.7. Collection and Screening of Environmental Data

To explore the possible effects of bioclimatic variables on the distribution suitability of snow leopards, we used the WorldClim database (https://www.worldclim.org/, accessed on 20 January 2022), which provides high-resolution climate data, as shown in (Table 1). A total of 19 bioclimatic variables were initially selected, derived from monthly temperature and rainfall values, and are more biologically meaningful [23] (Table 2).

**Figure 3:**
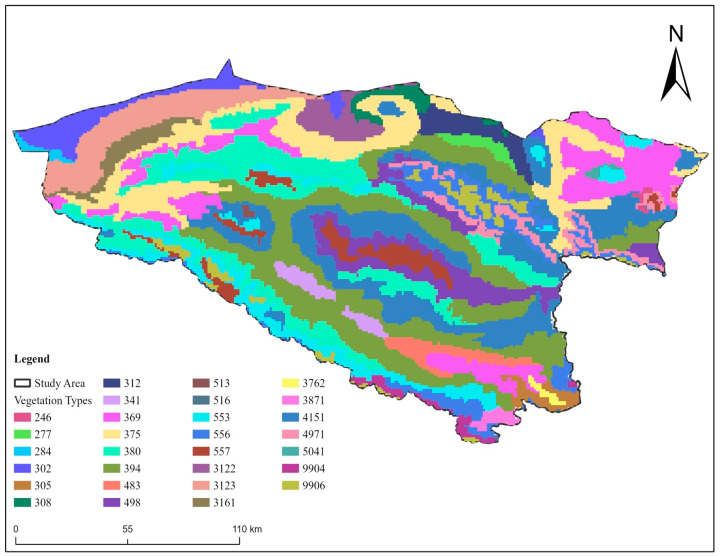
Map of the study area showing the distribution of various vegetation types. Each color represents a different vegetation type, coded numerically in the legend. The study area is outlined in black.

**Figure 4:**
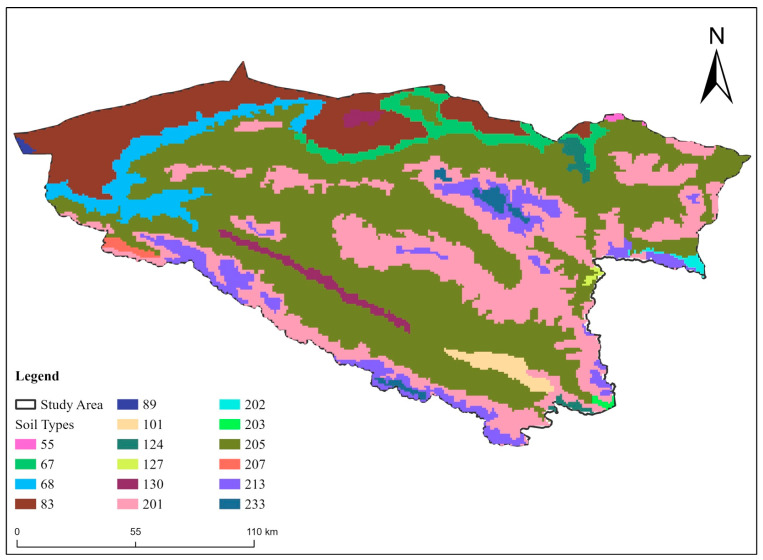
Map of the study area showing the distribution of various vegetation types. Each color represents a different vegetation type, coded numerically in the legend. The study area is outlined in black.

Additionally, data on Elevation; Altitude (m), vegetation type (shrub, forest, and grassland), soil type, human footprint, and land use were gathered from different sources to assess their impact on snow leopard distribution (Table 1 and Figure 3). The human footprint in the current study was assessed using a composite index that integrates different indicators of human presence and impact, including population density, road proximity, land use patterns, and infrastructure development. Each component was assigned a weight based on its perceived impact on snow leopard habitats, and these were summed to obtain a final footprint score. The units of measurement for the human footprint are indicated as an index score ranging from 0 to 3, where 0 represents no human impact and 3 indicates maximum human disturbance. This index allows for a standardized assessment of human influence across different regions, facilitating comparisons and trend analysis. We used the matching altitude data provided by SRTM (Shuttle Radar Topography Mission). The soil type and vegetation type data were obtained from the Resource and Environment Center of the Chinese Academy of Sciences (https://www.resdc.cn/, accessed on 15 February 2022), the human footprint data were obtained from the http://wcshumanfootprint.org (accessed on 15 February 2022) website database, the land use data was obtained, from the US land the satellite Landsat imagery was manually interpreted. To ensure that the modeling process is as accurate as possible, all environmental data were resampled in ArcGIS 10.8 software to ensure that the spatial resolution of all environmental data was 30 s (~1 km^2^) (Table 3 and Figure 4).

**Figure 5:**
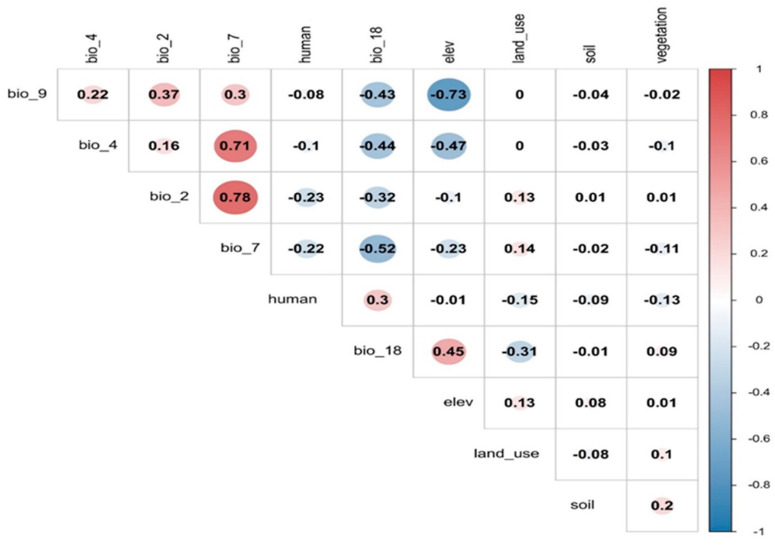
Correlation coefficients among various environmental variables used in the study, including bioclimatic factors, human footprint, elevation, land use, soil type, and vegetation. The color scale from blue to red indicates the strength and direction of correlations, with red representing a strong positive correlation and blue a strong negative correlation.

A correlation analysis was conducted to identify highly correlated variables. Variables with high correlation coefficients and those with a contribution rate of zero were removed, resulting in a final set of seven environmental factors for use in the MaxEnt model. Variables with a correlation coefficient greater than 0.75 were deemed strongly correlated and thus suitable for elimination to avoid multicollinearity. The cutoff value for exclusion based on correlation was established at 0.75, in line with conventional ecological modeling methods to ensure the input parameters’ robustness and independence [23] (Figure 5). In addition, variables with a contribution rate of zero in preliminary model runs were eliminated, leaving just a refined set of seven key environmental factors for final modeling.

### 2.8. Maximum Entropy Model Parameter Settings

We used the MaxEnt 3.4.1 software (https://biodiversityinformaics.amnh.org/open_source/maxent, accessed on 20 February 2022) to model the habitat suitability, employing a cross-validation method with ten repetitions and creating environmental factor response curves. We measured the importance of environmental variables via jack-knife test. The Maxent prediction results are output in Logistic format and saved in ASC format files. The result is rendered after converting to a TIF format file through ArcGIS 10.8. The receiver operating characteristic curve (ROC) method was used to test the model’s accuracy. The ROC curve is based on the model accuracy of non-threshold-independent evaluation, that is, each value of the predicted result is used as a possible judgment threshold, and the corresponding sensitivity and specificity are calculated from this. The AUC value is the ROC curve and the abscissa range. The area under the ROC curve can be used to describe the accuracy of the model simulation value. The ideal situation is that the predicted distribution area of the model completely matches the actual distribution area of the species, and the AUC value is 1 at this time. The evaluation standard of the ROC curve is as follows: if the AUC is between 0.8 and 0.9, the prediction result is considered fair; if the AUC is between 0.90 and 0.95, the prediction result is considered good; if the AUC is between 0.95 and 1.00, the prediction result is considered excellent [24].

## 3. Results

### 3.1. Habitat Suitability Distribution of Snow Leopards

**Figure 6:**
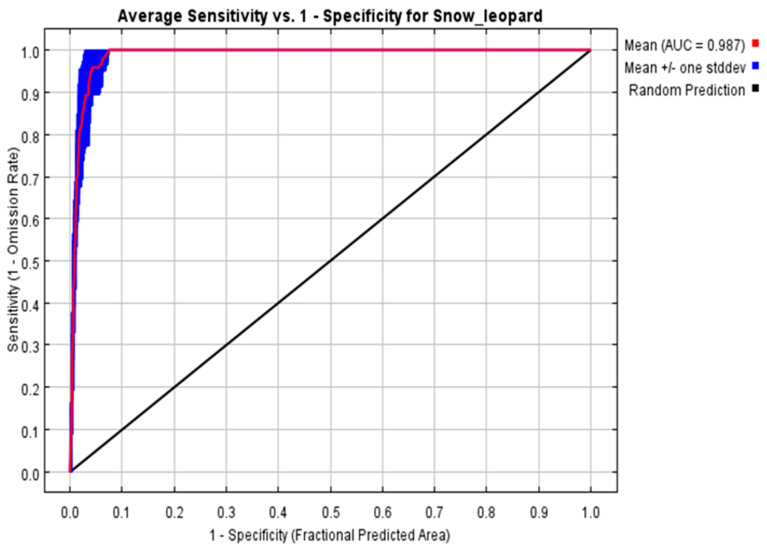
AUC values for the maximum entropy values.

**Figure 7:**
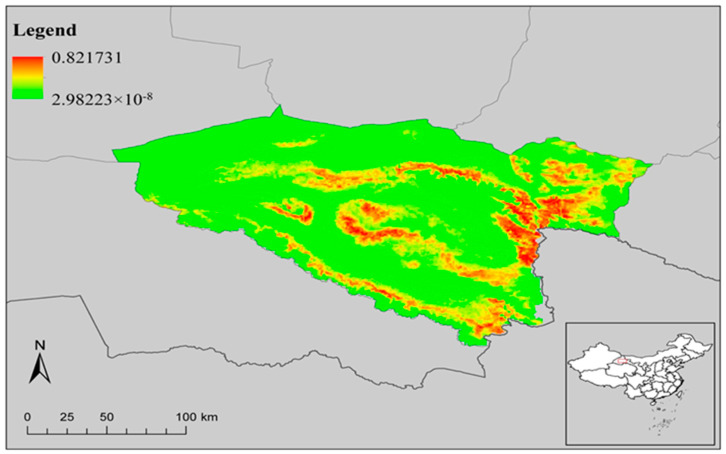
Spatial distribution of habitat suitability for snow leopards.

The AUC value of the maximum entropy model is 0.987 (Figure 6). This shows that the model prediction results are excellent and the reliability is high. According to the prediction results of the maximum entropy model, the most favorable environments are largely found around mountain ranges in the Yanchiwan National Nature Reserve, which correspond to places with lesser human activity. These high-suitability zones form band-like distributions that reflect the mountainous environment, emphasizing the snow leopards’ predilection for tough, less-accessible areas (Figure 7).

### 3.2. Environmental Drivers of Suitability Distribution of Snow Leopards

The jack-knife test indicates the relative importance of numerous environmental parameters in determining habitat suitability (Table 4). Notably, height and human footprint appear to be important influences, as does soil type. The response curves for these variables, particularly soil type and human activities, show a complex relationship with snow leopard distribution patterns.

**Figure 8:**
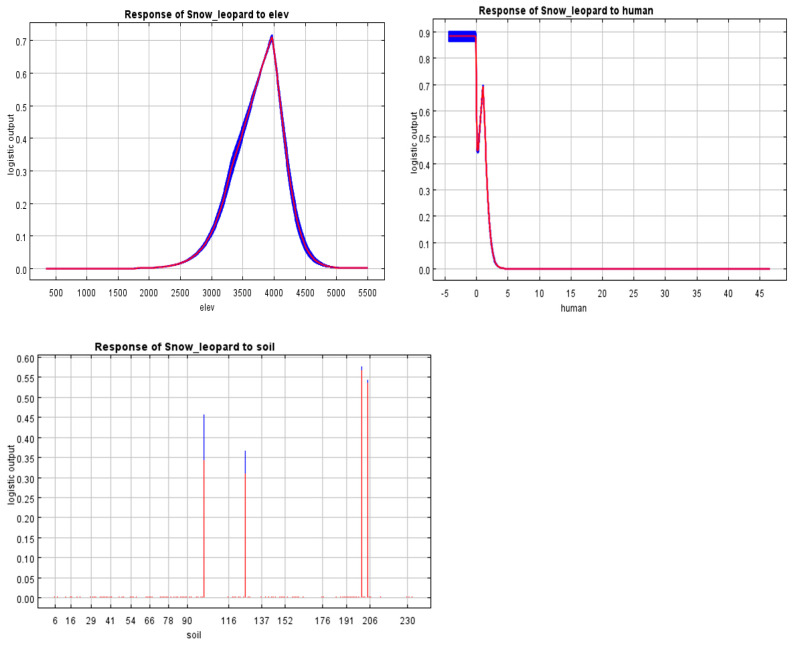
Response curves for environmental variables in snow leopard habitat.

Among them, altitude, human footprint, and soil type have a large contribution rate to the distribution of snow leopards. The response curves of these variables are shown in (Figure 8). In this study, soil type was considered a key environmental variable influencing snow leopard habitat suitability. The soil types were coded numerically to facilitate analysis, with each number on the *x*-axis of Figure 5 representing a unique soil class. These classes were derived from (https://www.resdc.cn/data.aspx?DATAID=145, accessed on 20 February 2022), which categorizes soil based on characteristics such as texture, composition, and hydrological properties. A detailed description of each soil type code and its corresponding soil class is provided to elucidate the impact on habitat suitability, ensuring clarity in the interpretation of model results.

**Figure 9:**
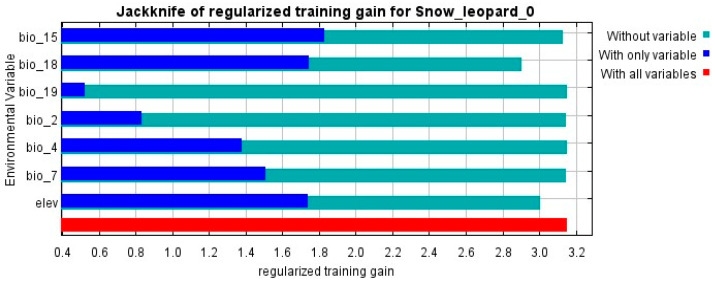
Jack-knife of regularized training gain for snow leopard habitat suitability model.

This indicates that among natural factors, altitude (29.2%) and soil type (23.9%) are the factors that have a greater impact on the distribution of snow leopard suitability. An altitude of about 3800 m is suitable for the distribution and activities of snow leopards, and the soil types that have a high impact on the distribution of snow leopards are mainly alpine soils. On the other hand, human recreational activities (such as climbing, hiking, and sports, as well as other forms of human presence and disturbance) accounted for 24.6% and had a significant impact on the distribution of habitat suitability for snow leopards (Figure 8). With frequent human recreational activities, the suitability of snow leopard habitats shows a sharp downward trend. The fluctuations in the middle may indicate areas where humans can access middle- and high-altitude regions, suggesting that human activities possibly intersect with the snow leopard’s habitat range. Our jack-knife analysis identifies the unique contributions of individual environmental factors to model correctness. Notably, when employed alone, soil type has the greatest positive impact on model gain, demonstrating its importance in forecasting snow leopard habitats. In contrast, omitting the human footprint data results in the greatest drop in model performance, highlighting its value from another perspective. These findings highlight the complicated and unique influence of these variables on snow leopard distribution, providing useful information for improving habitat suitability models (Figure 9).

### 3.3. Spatial Distribution Analysis for Snow Leopard Habitat Suitability

In addition to MaxEnt modeling, spatial distribution analysis was used to create a regionally detailed representation of habitat suitability within the Yanchiwan National Nature Reserve. MaxEnt estimates possible habitat compatibility using environmental variables and presence data, whereas spatial distribution analysis uses actual sightings and geographic data to identify present habitat conditions. This method improves our understanding by mapping the precise locations of high-suitability zones, providing useful information for field conservation activities. These findings are critical for on-the-ground management because they identify specific areas where conservation resources might be best targeted.

**Figure 10:**
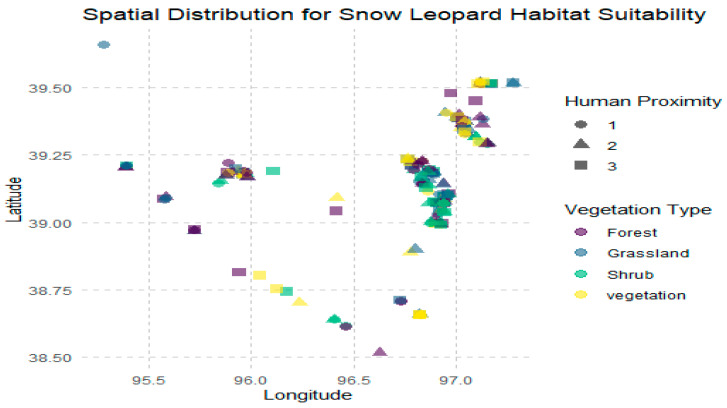
Spatial distribution of snow leopards in the study area, showing the areas of highest habitat suitability and potential human impact zones.

The spatial distribution analysis provided a crucial insight into snow leopard habitat suitability within the Yanchiwan National Nature Reserve. A wide range of geographic coordinates was observed, indicating a diverse range of habitats across the reserve. This distribution allowed for identifying specific hotspots where snow leopards are likely to thrive. These hotspots were characterized by higher altitudes, generally above 3300 m; sparse vegetation such as shrub and grassland; and lower levels of human proximity, suggesting minimal human disturbance. The findings suggest the snow leopards prefer mountainous regions with limited human activity, aligning with their known behaviors and habitat preferences (Figure 10).

### 3.4. Cluster Analysis for Snow Leopard Habitat Suitability

Cluster analysis was used to divide the research area into several habitat categories based on a variety of environmental parameters, including ones that MaxEnt does not explicitly analyze, such as local vegetation density and specific human impact assessments. This strategy offers a distinct perspective by identifying homogeneous habitat clusters that may react similarly to conservation initiatives. Unlike MaxEnt, which provides a continuous suitability gradient, cluster analysis breaks down habitat diversity into manageable units, making it easier to implement targeted conservation policies and allocate resources.

**Figure 11:**
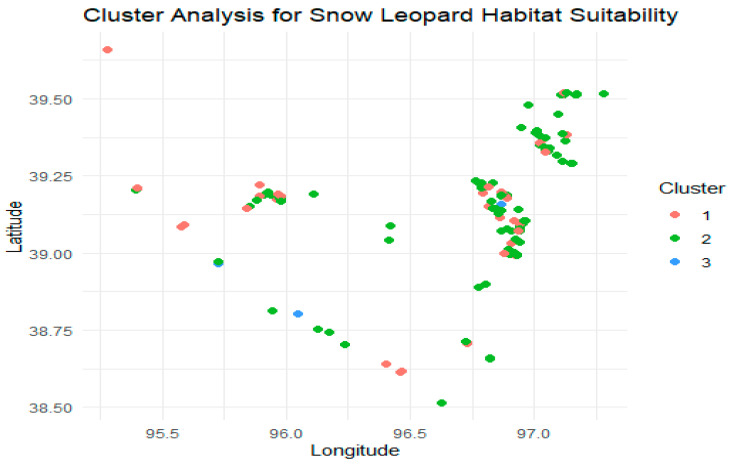
Cluster analysis of snow leopards in the study area.

The cluster analysis of the Yanchiwan National Nature Reserve revealed three distinct clusters, indicating varying habitat suitability for snow leopards. These clusters were formed based on environmental characteristics such as altitude, temperature, and human proximity. The analysis showed that areas with higher altitudes and lower human proximity tended to group together, suggesting that these conditions contribute to higher habitat suitability. The spatial distribution of the clusters indicated that certain regions in the reserve offer better conditions for snow leopards, primarily due to their reduced human impact and specific environmental traits. This finding is significant for conservation planning as it allows us to target specific clusters that are most conducive to snow leopard habitats. By understanding these distinct clusters, conservation efforts can be more effectively directed to preserve and protect the areas where snow leopards are likely to thrive (Figure 11).

### 3.5. Temporal Trends in Habitat Suitability for Snow Leopards

**Figure 12:**
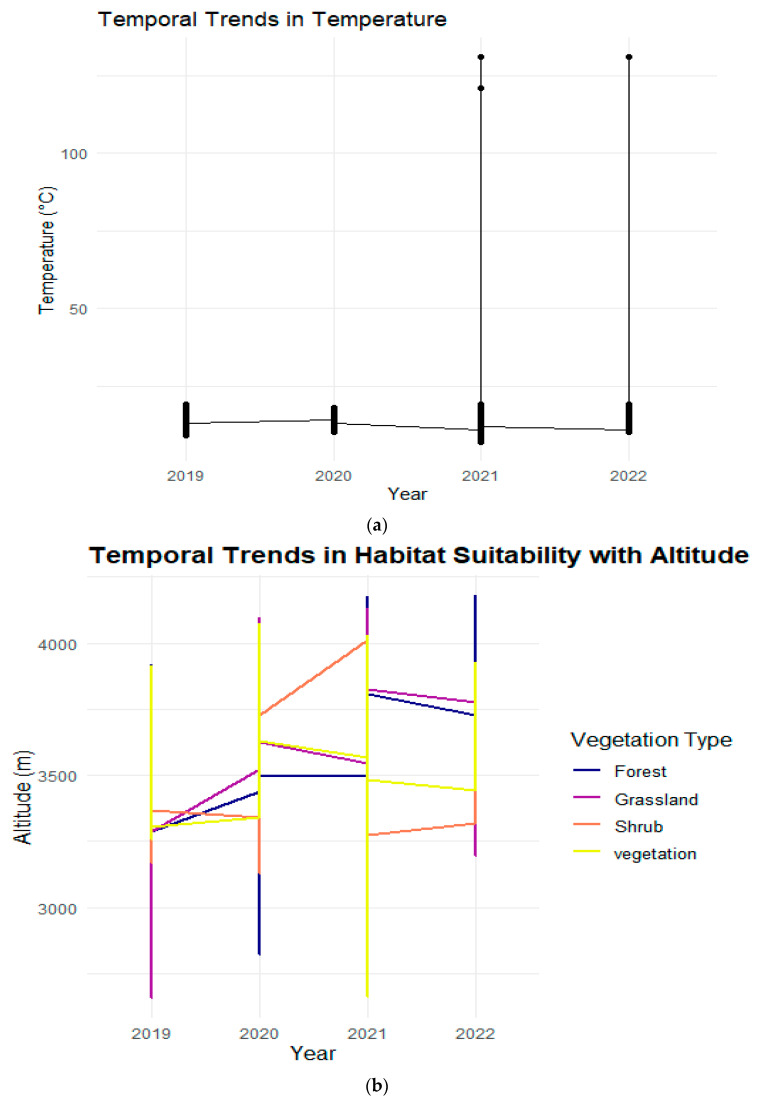

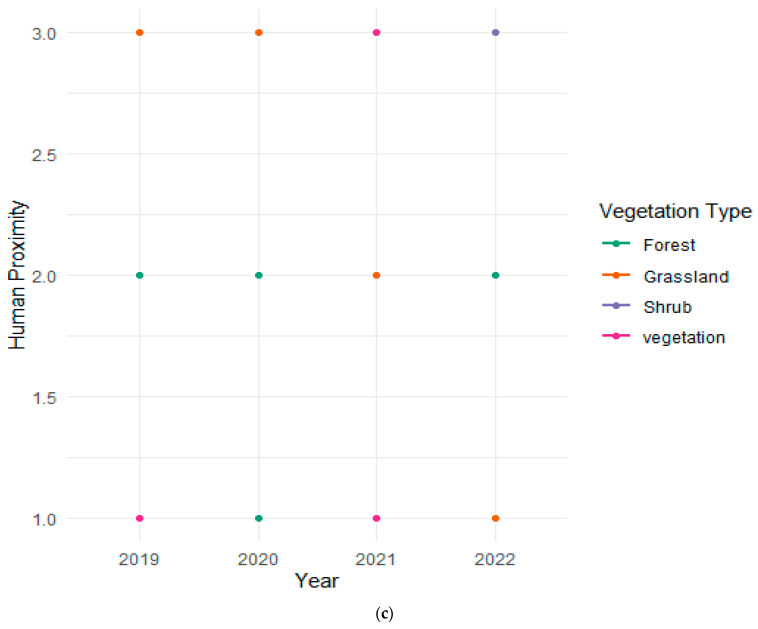
(**a**): Annual peak temperatures from 2019 to 2022. This graph illustrates the highest recorded temperatures in each year, reflecting annual climatic variations. (**b**): Yearly altitude range for different vegetation types from 2019 to 2022. This graph displays the altitude variations where different vegetation types are most prevalent, illustrating changes in vegetation distribution patterns over time. (**c**): Changes in human proximity to various vegetation types from 2019 to 2022. Each point indicates the average distance of human activities from areas dominated by specific vegetation types, highlighting potential impacts on habitat disturbances.

Our analysis highlighted noticeable fluctuations in temperature across different years within the reserve, reflecting significant variability in climatic conditions. The line plot of annual temperatures (Figure 12a) illustrates these fluctuations, emphasizing a pattern of change in the climatic baseline over the study period. Specifically, the data show a trend of increasing and decreasing temperatures from one year to the next, underscoring the dynamic nature of the environment in which snow leopards reside. The analysis revealed distinct patterns in habitat suitability concerning altitude for snow leopards. Across different vegetation types, we observed variations in the response of habitat suitability to changes in altitude over time. In general, higher altitudes tended to exhibit lower habitat suitability, particularly in areas dominated by shrubs and forests. Conversely, grasslands and certain types of vegetation showed more favorable habitat conditions at lower altitudes. These findings suggest a complex interplay between altitude, vegetation type, and habitat suitability for snow leopards, highlighting the need for comprehensive conservation strategies that consider these factors to ensure the protection of snow leopard habitats effectively. In examining the temporal trends of habitat suitability for snow leopards, we conducted an analysis focusing on the variable of human proximity within the habitat. Through visualizations generated using R programming, we observed a notable pattern indicating a correlation between human proximity and habitat suitability over time. Specifically, our findings reveal a concerning trend of decreasing habitat suitability as human proximity increases within the snow leopard habitat. This suggests a potential impact of human activities on the suitability of the environment for snow leopards, indicating a need for further investigation and conservation efforts to mitigate these effects and preserve suitable habitats for the species (Figure 12b,c)

## 4. Discussion

### 4.1. Impact of Environmental Factors on Snow Leopard Habitat

This study utilized the MaxEnt model to assess the influence of environmental conditions on snow leopard habitat suitability. Our finding is consistent with the results in previous research, emphasizing the importance of altitude, human footprint, and soil type in shaping snow leopard habitat. Studies such as Li et al. [14] and Zhang et al. [15] have documented similar influences on habitat suitability and distribution patterns for snow leopards across various regions [14,15]. MaxEnt was chosen due to its suitability for snow leopard distribution modeling, especially when dealing with presence-only data, as is often the case in wildlife studies [25].

While research has found some critical elements determining snow leopard habitats, these aspects can vary greatly between places. Our research contributes to this body of knowledge by presenting specific examples from the Yanchiwan National Nature Reserve, where varied soil compositions and human activity patterns alter habitat conditions.

Several studies have highlighted altitude as a major variable. Notably, Bai et al. [26] underlined its significance, but other studies, such as Qiao et al.’s [5] study in the Wolong National Nature Reserve, did not include altitude in their analysis, perhaps resulting in a missed opportunity to find habitat suitability characteristics. Furthermore, Bai et al. [26] reported on a comprehensive study at the Mount Everest National Nature Reserve that found that not only altitude but also temperature, terrain ruggedness, and yearly precipitation were important factors in snow leopard distribution. Furthermore, Xu Feng et al. [27] discovered that concealment caused by vegetation cover had a major impact on snow leopard habitat selection at nine sites, including the Altai and eastern Tianshan Mountains.

Altitude is an important factor in snow leopard habitats as it influences climate conditions, prey presence, and vegetation types, which are crucial for the species’ survival. Studies such as that by Sharma et al. [22] have demonstrated that snow leopards primarily occupy high-altitude regions where cooler temperatures and rugged terrain offer suitable conditions for their secretive nature and hunting practices.

Soil types also play a significant role, especially in terms of supporting prey species. Different soil types can support different vegetation structures, which ultimately affects the distribution of prey animals such as blue sheep and ibex. According to our findings, Alpine Meadow Soil and Mountain Brown Soil, which are generally found in high-altitude habitats, are critical for sustaining strong prey populations required by snow leopards. Research by Wang [21] underscores how soil characteristics influence prey habitats and, as a result, predator (snow leopards) distributions. According to our findings, the following soil types are important for snow leopard habitat: alpine meadow soil, mountain brown soil, and limestone soil. These soil types support a variety of flora that snow leopards rely on for hunting and cover. Alpine meadow soil and mountain brown soil, which are commonly found in high-altitude and mountainous areas, encourage the growth of grasses, tiny shrubs, and trees, providing valuable cover and hunting grounds. Limestone soil, which is abundant in karst locations, supports unique vegetation that contributes to the richness of snow leopard habitats. Despite the link between soil and vegetation types, our analysis revealed no significant direct effect of vegetation types on snow leopard distribution. This could be owing to snow leopards’ excellent adaptability to varied flora species, provided that other habitat criteria, such as prey availability and cover, are met. Furthermore, vegetation types may influence other elements, such as prey density and human disturbance, which are more important for snow leopard distribution. Furthermore, the scale at which vegetation data were studied may not accurately reflect snow leopards’ fine-scale habitat preferences. More comprehensive vegetation data at a finer spatial resolution may yield different conclusions. This is corroborated by studies such as those of Sharma et al. [22] and Li et al. [14], which highlight the complex connections between habitat elements that influence snow leopard distribution.

The human footprint is another key factor as it summarizes human activities and their impact on the natural environment. A low human footprint generally corresponds to less-disturbed habitats, which are crucial for the conservation of snow leopards. Our findings align with the broader literature, such as the work by Zhang et al. [19], which links increased human activities to higher stress levels and lower breeding rates in snow leopards. Furthermore, our analysis shows that the human footprint, which includes leisure activities such as hiking and climbing, has a considerable impact on snow leopard distribution by reducing habitat suitability in accessible places. This finding emphasizes the importance of regulating human activities within the reserve to reduce their influence on animals.

Since the suitable areas are recognized in our study, their adequacy for snow leopard conservation is evaluated based on several criteria: the size of the area, connectivity with other habitats, human disturbance levels, and prey availability. Our main findings indicate that while the suitable areas are extensive, their conservation efficacy depends upon maintaining connectivity and minimizing human disturbances.

On the grounds of our analysis and comparative studies, we find that isolated patches, even if ecologically suitable, are insufficient for long-term conservation unless integrated into a broader network of protected areas. This perspective is supported by findings from Janecka et al. [28], who highlight the importance of landscape connectivity in maintaining genetic diversity and ecological flexibility in snow leopard populations.

Additionally, in early surveys of snow leopards in Gansu and Qinghai, it was found that the main prey, such as the Himalayan marmot (*Marmota himalayana*) and the blue sheep (*Pseudois nayaur*), are also important factors affecting the distribution of snow leopards (Schaller et al., 1988 [7]). This is a factor considered in most studies on the habitat of snow leopards. Ecological relationships and dynamics between snow leopards and other species within their habitat have always been an important factor affecting species distribution, but few studies on species distribution models focus on such relationship [29]. The results of the study conducted in Qilian Mountain of Gansu Province show that the distribution of snow leopards is mainly affected by prey and altitude [30]. With the increase in human recreational activities, the habitat changes of these snow leopards’ main prey are also worthy of attention in the future. The habitat changes of these snow leopards are likely to have a very significant impact on the snow leopard habitat.

### 4.2. Limitations of the Study

While our study aimed to assess snow leopard habitat suitability based on occurrences, several limitations need to be acknowledged. First, the elusive behavior of snow leopards and their vast home ranges pose significant challenges to population size estimation. As noted by Sharma et al. [22], conducting comprehensive population surveys for snow leopards is inherently difficult and costly due to their secretive nature and preference for rugged terrains. Consequently, our study relied on habitat suitability assessments rather than direct population counts to infer snow leopard presence.

Furthermore, the lack of systematic surveys covering large areas and the limited availability of long-term monitoring data contribute to uncertainties regarding snow leopard distribution and population dynamics. While our research scope encompassed specific areas within the Yanchi Bay Reserve, the absence of comprehensive, long-term field monitoring hinders the development of a holistic understanding of snow leopard populations. Jackson et al. [1] underscores the importance of sustained monitoring efforts for accurate population status assessments and conservation decision-making.

Additionally, our study did not incorporate detailed information on prey species distribution, which is a crucial determinant of snow leopard habitat suitability. As acknowledged by Sharma et al. [22], the availability of prey species directly influences snow leopard habitat selection and occupancy patterns. Future research should aim to integrate prey distribution data to enhance the robustness of habitat suitability assessments.

Moreover, the climate data utilized in our study were sourced from the WordClim database, which may have spatial resolution limitations for research at the scale of protected areas. While bioclimatic variables are widely used in species distribution modeling, localized climate data would provide more precise environmental predictors for snow leopard habitat suitability assessments. Improved data monitoring and availability are essential for refining species distribution models and guiding conservation efforts effectively.

In conclusion, while our study provides valuable insights into snow leopard habitat suitability, the aforementioned limitations underscore the need for further research incorporating population size estimation, long-term monitoring, prey distribution data, and refined climate data to enhance the accuracy and applicability of conservation strategies for this endangered species.

## 5. Recommendations

The results of this study show that human footprint will affect the selection and utilization of snow leopard habitats. The rapid development of China’s social economy and expansion of infrastructure have had a negative impact on snow leopards and the entire alpine ecosystem [31]. Therefore, in the process of planning and implementing infrastructure construction in this area, it is necessary to consider the potential suitable habitat for snow leopards, comprehensively evaluate the conditions, and fully carry out the planning of protected areas and the core protected areas through suitability evaluation method [32].

Local management departments should strictly adhere to policy requirements to eliminate grazing, poaching, and illegal mining and selling. They should aim to maximize the protection of rare and endangered wild animals, such as snow leopards, through scientific management and effective measures. At the same time, based on the existing research, a more comprehensive survey of snow leopard distribution should be conducted in Yanchi Bay Reserve, and more detailed environmental data should be recorded in the survey to explore the ecosystem structure of snow leopard survival. To effectively understand and manage the factors influencing snow leopard habitat selection, it is essential to construct ecological corridors for the long-term survival, development, and migration of adult snow leopard populations in the region as soon as possible. Additionally, we must restore and rebuild damaged habitats [28].

Finally, a comprehensive snow leopard monitoring system must be properly created. For example, through the combination of remote sensing technology and using surface ecological monitoring methods to monitor continuously and evaluate the quality of the ecological environment in mountainous areas for a long time and record its dynamic evolution, building a data-sharing network in the entire mountainous area is beneficial to grasping the overall ecological environment status and comprehensively grasping the distribution of snow leopards, solving the current problem of incomplete sampling, and fully ensuring timely feedback on the overall ecological health of the mountainous area.

## 6. Conclusions

This study used the MaxEnt model to estimate snow leopard habitat appropriateness in the Yanchiwan National Nature Reserve based on field surveys conducted from 2019 to 2022. Key findings show that height, human activity, and soil type have the greatest influence on habitat suitability, with the most favorable places being found along mountain ranges. This analysis identifies key regions that require immediate conservation actions in order to reduce human impact and habitat fragmentation. These findings are critical for directing local government initiatives in Gansu Province, China, in protected area planning and biodiversity protection. Furthermore, the approach and findings of this study serve as a model for similar conservation efforts around the world, particularly in areas where data are limited and human–wildlife conflicts are prevalent. This research not only improves our understanding of snow leopard habitats; it also helps focused conservation measures, which contribute to the long-term survival of snow leopards in their native context.

## Figures and Tables

**Table 1:** Distribution of soil types in the Yanchiwan National Nature Reserve. The table illustrates the count and percentage of each soil type observed, providing insights into the predominant environments within the study area.

No.	Soil Types Code	Soil Types	Percentage
1	55	Chestnut Calcic Soil	0.08%
2	67	Brown Calcic Soil	2.98%
3	68	Light Brown Calcic Soil	4.36%
4	83	Grey Brown Desert Soil	11.84%
5	89	Gypsum Brown Desert Soil	0.10%
6	101	Steppe Wind Sandy Soil	1.09%
7	124	Calcareous Coarse Skeletal Soil	0.66%
8	127	Calcareous Meadow Soil	0.10%
9	130	Salinized Meadow Soil	1.43%
10	201	Cold Calcic Soil	22.39%
11	202	Dark Cold Calcic Soil	0.24%
12	203	Light Cold Calcic Soil	0.10%
13	205	Cold Calcic Soil	48.24%
14	207	Light Cold Calcic Soil	0.29%
15	213	Frozen Soil	5.33%
16	233	Glacial Snow Cover	0.77%

**Table 2:** Maximum entropy modeling of environmental factors, listing the bioclimatic variables used in the study along with other environmental factors such as elevation, soil type, vegetation type, human footprint, and land use.

Abbreviations	Environmental Factor
BIO1	Annual Mean Temperature
BIO2	Mean Diurnal Range (mean of monthly (max temp − min temp))
BIO3	Isothermality (BIO2/BIO7) (×100)
BIO4	Temperature Seasonality (standard deviation × 100)
BIO5	Max Temperature of Warmest Month
BIO6	Min Temperature of Coldest Month
BIO7	Temperature Annual Range (BIO5 − BIO6)
BIO8	Mean Temperature of Wettest Quarter
BIO9	Mean Temperature of Driest Quarter
BIO10	Mean Temperature of Warmest Quarter
BIO11	Mean Temperature of Coldest Quarter
BIO12	Annual Precipitation
BIO13	Precipitation of Wettest Month
BIO14	Precipitation of Driest Month
BIO15	Precipitation Seasonality (coefficient of variation)
BIO16	Precipitation of Wettest Quarter
BIO17	Precipitation of Driest Quarter
BIO18	Precipitation of Warmest Quarter
BIO19	Precipitation of Coldest Quarter
Elev	Elevation
Soil	Soil type
Vegetation	Vegetation type
Human	Human footprint
Land_use	Land Use

**Table 3:** Distribution of vegetation types in the Yanchiwan National Nature Reserve. The table illustrates the count and percentage of each vegetation type observed, providing in-sights into the predominant environments within the study area.

No.	Vegetable Types Code	Vegetable Types	Percentage
1	246	Subalpine Deciduous Broadleaf Shrubland	0.14%
2	277	Temperate Shrub Desert	0.94%
3	302	Temperate Semi-shrubs and Dwarf Semi-shrubs Desert	14.25%
4	341	Temperate Succulent Halophytic Dwarf Semi-shrubs Desert	1.08%
5	369	Temperate Tufted Grass Typical Steppe	30.52%
6	394	Temperate Tufted Dwarf Grass and Dwarf Semi-shrubs Desert Steppe	16.53%
7	4151	Alpine Grass and Sedge Steppe	12.95%
8	483	Temperate Grass and Miscellaneous Grass Halophytic Meadow	1.41%
9	498	Alpine Kobresia Grass and Miscellaneous Grass Meadow	7.65%
10	553	Alpine Cushion Vegetation	5.34%
11	556	Alpine Sparse Vegetation	6.76%
12	9904	Others	2.43%

**Table 4:** Contribution of environmental factors.

Variable	Percent Contribution (%)
Elev	29.2
Human	24.6
Soil	23.9
Bio_18	5.7
Bio_15	4.0
Bio_9	3.7
Vegetation	3.2
Bio_7	2.6
Bio_2	1.9
Land_use	0.7
Bio_4	0.5

## Data Availability

The data presented in this study are available on request from the corresponding author. The data are not publicly available due to privacy and ethical restrictions to protect the confidentiality of the study participants.
